# Special Issue “Vitamin D Signaling in Human Health and Diseases”

**DOI:** 10.3390/ijms27219463

**Published:** 2026-10-23

**Authors:** Daniela Rovito

**Affiliations:** Institut de Génétique et de Biologie Moléculaire et Cellulaire (IGBMC), CNRS UMR 7104, INSERM U1258, Université de Strasbourg, F-67400 Illkirch, France; rovitod@igbmc.fr

Few molecules have traveled as far from their textbook role as vitamin D. For more than a century, the so-called vitamin of the skeleton, it is now understood as a hormone whose nuclear receptor is wired into the transcriptional and epigenetic control of immune, metabolic and musculoskeletal tissues [1,2]. The question is no longer whether vitamin D acts beyond bone, but where in whom and at what dose its signaling can be exploited.

Vitamin D is a secosteroid hormone obtained mainly through cutaneous synthesis and, to a lesser extent, from the diet. In the skin, ultraviolet B (UVB) radiation converts 7-dehydrocholesterol into previtamin D_3_, which isomerizes to vitamin D_3_ (cholecalciferol). Dietary sources provide vitamin D_3_ from animal foods such as fatty fish, fish liver oils, and egg yolk, and vitamin D_2_ (ergocalciferol) from mushrooms, particularly when exposed to UV light, while fortified foods may contain either form [1]. Both forms are biologically inactive and circulate bound to the vitamin D-binding protein until they are activated by two sequential hydroxylations. Vitamin D is first hydroxylated in the liver, mainly by CYP2R1 and to a lesser extent by CYP27A1, to 25-hydroxyvitamin D (25(OH)D), the main circulating form and the standard biomarker of vitamin D status, and then, in the kidney, by the 1α-hydroxylase CYP27B1 to 1,25-dihydroxyvitamin D (calcitriol), its bioactive form [1]. Both metabolites are inactivated by the 24-hydroxylase CYP24A1, which provides the main catabolic pathway [1]. Calcitriol acts mainly through the vitamin D receptor (VDR), a ligand-activated nuclear receptor that heterodimerizes with the retinoid X receptor alpha (RXRA) and binds vitamin D response elements in target genes [3,4].

However, this canonical view has been expanded by recent studies. The steroidogenic enzyme CYP11A1, best known for converting cholesterol into pregnenolone, can also act on vitamin D_3_ and initiate an alternative pathway of activation, hydroxylating the side chain mainly at C20 to generate 20-hydroxyvitamin D_3_ and 20,23-dihydroxyvitamin D_3_, which can be further modified by CYP27B1 and CYP24A1 and have been detected in human tissues [5,6]. The same enzyme also metabolizes 7-dehydrocholesterol and the lumisterol and tachysterol photoproducts of ultraviolet B exposure, expanding the repertoire of secosteroids and related sterols with biological activity [7,8]. In preclinical studies, these derivatives are largely non-calcemic and act on the VDR, but they also engage other receptors. They act as inverse agonists on the retinoic acid-related orphan receptors RORα and RORγ and have been reported to signal through the aryl hydrocarbon receptor (AhR) and the liver X receptors (LXRs) [8,9,10]. Most of this evidence derives from in vitro and animal models, and its physiological relevance in humans remains to be established. This suggests that vitamin D biology is better described as a network of metabolites, receptors, and transcriptional partners than as a single ligand-receptor axis, in which serum 25(OH)D captures only part of the system.

Despite this broad biological reach, translation into clinical practice remains challenging. Deficiency is common worldwide [11], but large supplementation trials have often shown modest or inconsistent benefits [12], and current guidelines restrict empiric supplementation to specific populations [13]. Part of this inconsistency reflects the fact that responses vary with genetic background, baseline vitamin D status, and tissue context [14]. Beyond supplementation, pharmacological modulation of VDR is constrained by hypercalcemia, which limits the doses of calcitriol and its analogs that can be safely used [15], and by the absence of clinically available VDR antagonists. This Special Issue was conceived to gather research articles and reviews on the molecular pathways, cellular responses, and clinical outcomes of vitamin D signaling. It includes five papers: two original articles and three reviews.

Together, these contributions can be read as a path from the gene to the clinic. Atopic dermatitis is a common chronic inflammatory skin disease [16], and lower serum 25(OH)D has been associated with greater severity, while supplementation may improve symptoms [17]. At the level of the gene, Briedė et al. [Contribution 1] studied adults with atopic dermatitis, a population in which data on VDR polymorphisms are scarce, in a high-latitude country where vitamin D insufficiency is common. Several of the variants they analyzed lie in the 3′ region of the VDR gene and are thought to influence mRNA stability rather than protein structure [18]. Genotyping six VDR polymorphisms in 91 patients and 102 controls, they found no differences in genotype or allele frequencies, but within the patient group the rs731236 G and rs1544410 T alleles were associated with higher odds of serum 25(OH)D above 30 ng/mL, the rs7975232 C allele with lower odds, and the rs11168293 T allele with eosinophil counts above 300 cells/μL. These nominal associations did not survive correction for multiple testing, and the authors suggest that VDR variation may modulate disease-related biomarkers rather than susceptibility in Lithuanian adults. Popov et al. [Contribution 2] started from genome-wide data showing that VDR/RXRA composite elements are found in only 11.5% of VDR ChIP-seq peaks in human monocytes [19] and hypothesized that VDR also cooperates with other transcription factors at genes linked to atopic and respiratory diseases. In myeloid cells, VDR directly controls immune genes such as the antimicrobial peptide cathelicidin [20], and meta-analyses of randomized trials suggest that vitamin D supplementation may reduce asthma exacerbations [21]. Combining motif analysis, AlphaFold 3 modeling of protein complexes [22], and expression studies in calcitriol-treated U937 macrophage-like cells, the authors predicted novel VDR/NR2C2 and VDR/PPARG composite elements in immune genes, with IRF5 showing the strongest response and a putative synergism between VDR/NR2C2 and VDR/RXRA elements. Binding was not directly demonstrated, but the work suggests that the repertoire of VDR-regulated genes is still incomplete.

These regulatory layers extend to the epigenome. Epigenetic alterations are recognized hallmarks of aging [23], and VDR depends on pioneer factors such as CEBPA to access chromatin and shape cell-type-specific responses [24]. Guo et al. [Contribution 3] review how VDR signaling may counteract age-related epigenetic alterations in immune, musculoskeletal, and nervous tissues, emphasizing that most mechanistic evidence comes from in vitro and preclinical models. In humans, the original DO-HEALTH analysis found that omega-3 supplementation slowed several epigenetic clocks by 2.9–3.8 months over three years, with an additive effect of omega-3, vitamin D and exercise on PhenoAge only, whereas vitamin D alone had no significant effect [25]. Guo et al. conclude that vitamin D monotherapy offers limited benefit for aging and advocate serum-monitored, individually titrated and multimodal strategies, with supplementation reserved mainly for documented deficiency.

The same tension between mechanism and clinical proof emerges in metabolic and muscular settings. Fuentes-Barría et al. [Contribution 4] discuss metabolic dysfunction-associated steatotic liver disease (MASLD), a highly prevalent condition with limited pharmacological options, in which VDR signaling intersects with insulin signaling, AMPK-mTOR energy sensing, inflammation and the liver-adipose axis. Clinical results remain heterogeneous because of differences in study design, assays, and 25(OH)D thresholds, and no major hepatology or metabolic society recommends routine supplementation outside confirmed deficiency; the authors therefore see vitamin D as an adjunctive rather than primary therapy, in line with current guidance [13]. Finally, Valle et al. [Contribution 5] focus on Down syndrome, one of the most common chromosomal conditions [26], where frequent vitamin D deficiency, which can persist in children despite supplementation [27], coexists with hypotonia, excess body weight, immune dysregulation and accelerated sarcopenia. They propose composite biomarker panels, combining 25(OH)D with cytokines, adipokines, myokines and functional muscle measures, to guide personalized interventions, while acknowledging that optimal dosing and many mechanistic links remain to be established in this population.

Despite their diversity, these contributions converge on a common message: the biological effects of vitamin D depend on genetic background, baseline vitamin D status, tissue context and the transcriptional partners of VDR, and possibly on the alternative metabolites and receptors that extend the classical vitamin D axis. They also point to two often-overlooked aspects. First, low 25(OH)D may be a consequence as well as a cause of disease [28], as in MASLD, where hepatic dysfunction and adipose sequestration lower circulating levels [Contribution 4]. Second, vitamin D catabolism through CYP24A1 contributes to interindividual variability in the response to supplementation [Contributions 3 and 4], as shown by genetic variants in CYP24A1 and related genes that modify the rise in 25(OH)D after supplementation [29]. Several authors call for individually titrated, biomarker-guided strategies rather than uniform supplementation and for long-term, well-designed trials that integrate mechanistic insight with clinical outcomes and lifestyle interventions [Contributions 3–5]. The call for papers also invited contributions on bone health, cardiovascular function and cancer prevention. Bone and cartilage are addressed in the review by Guo et al. [Contribution 3], who point out that large trials have not shown a reduction in fracture risk with vitamin D alone, without calcium, in individuals with adequate baseline status [30]. Cardiovascular function and cancer prevention were not addressed as dedicated topics and remain open opportunities for future work, all the more so as large trials have not shown a clear benefit of vitamin D in the general population [12].

Conclusions: This Special Issue illustrates both the scope of vitamin D signaling and the distance that still separates mechanistic insights from clinical practice. Future progress will likely depend on three complementary directions: mapping the genomic and epigenomic targets of VDR, and the contribution of alternative metabolites and receptors such as RORs, AhR and LXRs, in disease-relevant cells and tissues; stratifying individuals according to genetic background, baseline vitamin D status and catabolic capacity rather than treating them as a homogeneous population; and developing tissue-selective VDR ligands that dissociate therapeutic effects from calcemic activity [15]. Answering where in whom and at what dose vitamin D signaling can be targeted will require this convergence of molecular, clinical and population-level evidence.
